# Associations of daily sleep duration and dietary macronutrient consumption with obesity and dyslipidemia in Koreans: A cross-sectional study

**DOI:** 10.1097/MD.0000000000005360

**Published:** 2016-11-11

**Authors:** Hyungie Doo, Hyejin Chun, Miae Doo

**Affiliations:** a Department of Natural and Forensic Sciences, Albany State University, Albany, GA, USA; b Department of Family Medicine, Bundang CHA Medical Center, CHA University, Yatap-ro, Gyeonggi-do; c Department of Nutritional Science and Food Management, Ewha Womans University, Ewhayeodae-gil, Seoul, Republic of Korea.

**Keywords:** BMI, dietary carbohydrate, dietary fat, obesity, sleep duration

## Abstract

Daily sleep duration is known to be associated with obesity and dyslipidemia.

This study was performed to examine the interactions between daily sleep duration and the risks of obesity and dyslipidemia according to dietary macronutrient consumption in 14,680 Korean adults using the 5th Korean National Health and Nutrition Examination Survey.

Sleep duration was inversely associated with body mass index (*P* < 0.001), waist circumference (*P* < 0.001), total cholesterol (*P* < 0.001), and low-density lipoprotein -cholesterol (*P* = 0.001). Participants with short sleep durations consumed less dietary protein (*P* < 0.001) and fat (*P* < 0.001), and consumed more dietary carbohydrates (*P* < 0.001). Among participants with the shortest sleep duration (≤5 hours a day), the odds ratio of obesity was found to increase in the high fat consumption group (1.393, 95% confidence interval 1.083–1.790) and decrease in the high carbohydrate consumption group (0.770, 95% confidence interval 0.604–0.983). High fat and low carbohydrate consumption were confirmed to be associated with the risk of obesity in the shortest sleep duration group (≤5 hours a day).

These findings indicate that sleep duration was negatively associated with obesity and dyslipidemia-related indices in Korean adults. Additionally, the association of short sleep duration with the risk of obesity was potentially changed by dietary fat and carbohydrate consumption.

## 1 Introduction

Insufficient durations of sleep have been reported to be related with cardiometabolic problems,^[1]^ including obesity,^[2,3]^ type 2 diabetes mellitus,^[3,4]^ dyslipidemia,^[5,6]^ and cardiovascular disease.^[3,7]^ Accordingly, there has been much interest in sleep duration and cardiometabolic profiles. Many studies^[1–5,7]^ have reported that short and long sleep durations are associated with adverse health outcomes in a U-shaped pattern; however, other studies have not shown this pattern of association.^[6,8]^

The excessive fat accumulation caused by obesity is likely to change lipid metabolism, that is, obesity can lead to the development of dyslipidemia.^[9]^ Dyslipidemia caused by obesity is influenced by lifestyle factors, including diet and physical activity, in addition to genetic factors. Dietary factors, such as total energy and dietary compositions, are very important contributors in the etiology of obesity and dyslipidemia.^[10]^ However, modifying dietary factors alone is insufficient to improve obesity and dyslipidemia, as insufficient sleep duration might be an additional factor influencing the development of these diseases.^[11–12]^

Although the associations of sleep duration with obesity and dyslipidemia are well-established, the definition of the appropriate duration of sleep for the prevention or improvement of obesity and dyslipidemia remains unclear. Additionally, as described above, modifications of these associations by diet have not been clearly elucidated. Therefore, the aim of this study was to examine the association of sleep duration (3 categories) with obesity and dyslipidemia-related indices using data from the 5th Korean National Health and Nutrition Examination Survey (KNHANES). Moreover, this study examined the interaction between sleep duration and dietary consumption in relation to the risks of obesity and dyslipidemia.

## 2 Subjects and methods

### 2.1 Study design and participant selection

**Figure 1:**
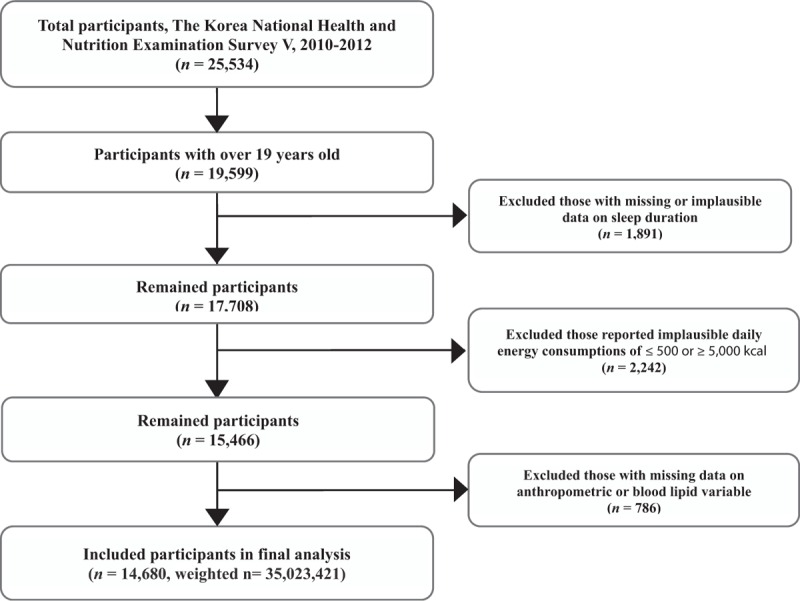
Framework of participant selection.

This study used data that were collected from the 5th KNHANES (2010–2012), which is a national cross-sectional survey that has been conducted annually by the Korean Centers for Disease Control and Prevention (KCDC) since 1998.^[13]^ The KNHANES consists of health interviews, health examinations (ie, a physical examination, laboratory test, and radiologic study), and dietary surveys including the frequency and type of food intake. The dietary surveys are conducted to investigate the relationship between health and the nutritional status of the population. The participants in the 5th KNHANES were selected from stratified multistage samples of the noninstitutionalized civilian South Korean population, and included a total of 25,534 people. Of these participants, 14,680 (weighted n = 35,023,421 including 17,430,498 men and 17,592,922 women) who were aged ≥19 years with a daily energy consumption >500 kcal and <5000 kcal, and had no missing data were enrolled in this study (Fig. 1). The survey protocol was approved by the KCDC Institutional Review Board (IRB No. 2010–02CON-21-C, 2011–02CON-06-C, 2012–01EXP-01–2C), and all participants signed an informed consent form to participate in the KNHANES. However, this study did not require any ethics approval, because the KNHANES data are publicly available.

### 2.2 Data collection

The participants’ age, sex, sleep duration, anthropometric measurements, and blood lipid profile data were collected through health interviews and health examinations.

Sleep duration was assessed using the following self-reported question: “How long do you usually sleep per day?” The reported duration was divided into 3 categories: ≤5 hours, 6 to 7 hours, and ≥8 hours of sleep per day. Duration of 6 to 7 hours per day was selected as the reference according to the average sleep duration identified in previous studies.^[14,15]^

The participants’ heights were measured to within 0.1 cm with an anthropometer, and their weights were measured using a digital weight scale to the nearest 0.01 kg. Body mass index (BMI) was calculated by dividing the weight (kg) by the square of height (m^2^), and BMIs greater than 25 kg/m^2^ were used to define obesity based on the recommendations of the Korean Society for the Study of Obesity.^[16]^ Waist circumference (WC) was measured to the nearest 0.1 cm at the midpoint between the lower border of the rib cage and the iliac crest at the end of a normal expiration using a non-elastic tape.

Blood lipid profiles were analyzed using venous blood samples that were collected in the morning after an overnight fast. Total cholesterol (TC), high-density lipoprotein cholesterol (HDL-C), and triglycerides (TGs) were measured using a Hitachi automatic analyzer 7600 (Hitachi High-Technologies, Tokyo, Japan). Furthermore, low-density lipoprotein cholesterol (LDL-C) concentrations were calculated using the equation described by Friedewald for individuals with TG concentrations <400 mg/dL.^[17]^ Dyslipidemia was defined by the Korean Society of Lipidology and Atherosclerosis criteria as exceeding 1 or more of the following cut-offs^[18]^: TC level >200 mg/dL, TG level >200 mg/dL, HDL-C level <40 mg/dL, or LDL-C level <130 mg/dL. These cut-offs differ from those of the modified National Cholesterol Education Program Adult Treatment Panel (NCEP ATP) III.

Information on the participants’ dietary consumption was obtained from the dietary surveys. Dietary consumption was assessed using a food frequency questionnaire that was developed and validated for the KNHANES.^[19]^ The cut-offs for the total daily energy consumption levels and percentages of the total daily energy from protein, fat, and carbohydrates (CHO) were defined by the medians (energy: 1830.00 kcal, protein: 13.63%, fat: 15.86%, and CHO: 69.72%).

### 2.3 Statistical analysis

Sleep durations were divided into tertiles based on the hours of sleep per day as follows: ≤5 h/d, 6 to 7 h/d, and ≥8 h/d. The analyses were conducted according to sex or sleep duration; *chi-square* tests were used to analyze the categorical variables, and *t* tests or analyses of variance (ANOVAs) were used to analyze the continuous variables. A multivariable logistic regression analysis was used to determine the odds ratios (ORs) and 95% confidence intervals (CIs) of obesity and dyslipidemia according to the interaction between sleep duration and dietary consumption after adjusting for age and sex. All statistical analyses were performed using the survey procedures of SPSS (version 21.0; IBM Corporation, Armonk, NY) software for Windows in accordance with the KNHANES complex sampling design. *P* values less than 0.05 were considered statistically significant.

## 3 Results

### 3.1 General characteristics

The characteristics of the participants including sleep duration, anthropometric measurements, blood lipid profiles, and dietary consumption are presented according to sex in Table 1. A total of 14,680 subjects [5,919 men (40.3%) and 8761 women (59.7%)] participated in this study. The average age was 44.26 ± 0.30 years for men and 45.75 ± 0.28 years for women (*P* < 0.001). The anthropometric measurements such as height, weight, BMI, and WC were significantly higher in men than in women (*P* < 0.001 for all). TC (*P* = 0.013) and TG (*P* < 0.001) levels were significantly higher in men; in contrast, HDL-C (*P* < 0.001) and LDL-C (*P* < 0.001) were significantly higher in women. However, there was no significant difference between men and women in sleep duration (6.87 ± 0.02 hours for men and 6.84 ± 0.02 hours for women; *P* = 0.250). Dietary consumption varied significantly according to sex; men consumed more energy (*P* < 0.001), protein (*P* < 0.001), and fat (*P* = 0.005), and less CHO (*P* < 0.001) than women.

**Table 1:**
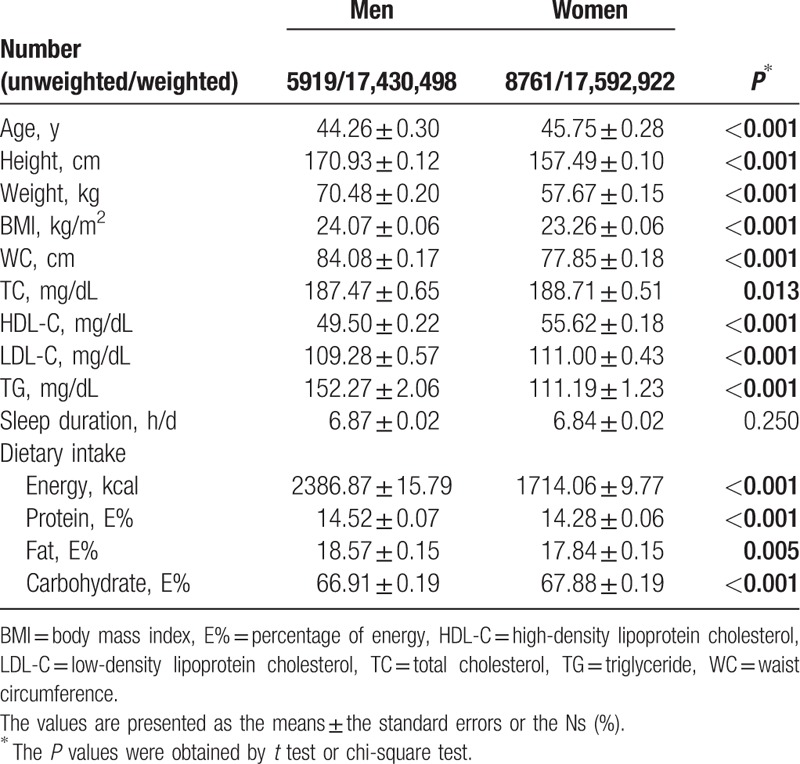
Anthropometric, blood lipid and dietary consumption of a Korean population.

### 3.2 Anthropometrics, blood lipid profiles, and dietary macronutrient consumption by sleep duration

Table 2 illustrates the anthropometric, blood lipid, and dietary macronutrient consumption data according to daily sleep duration. Of the total 14,680 subjects, 10.4% of men and 15.2% of women confirmed having a short sleep duration (≤5 hours a day). Subjects with short sleep durations were older (*P* < 0.001). Moreover, BMI (*P* < 0.001), WC (*P* < 0.001), TC (*P* < 0.001), and LDL-C (*P* = 0.001) were inversely associated with sleep duration. However, there was no significant difference in HDL-C or TG. The participants who slept ≥8 hours a day had lower BMIs and WC, TC, and LDL-C levels than those who slept ≤5 hours a day. Dietary energy consumption was significantly associated with sleep duration (*P* < 0.001), but the relationship was not proportional or inverse.

**Table 2:**
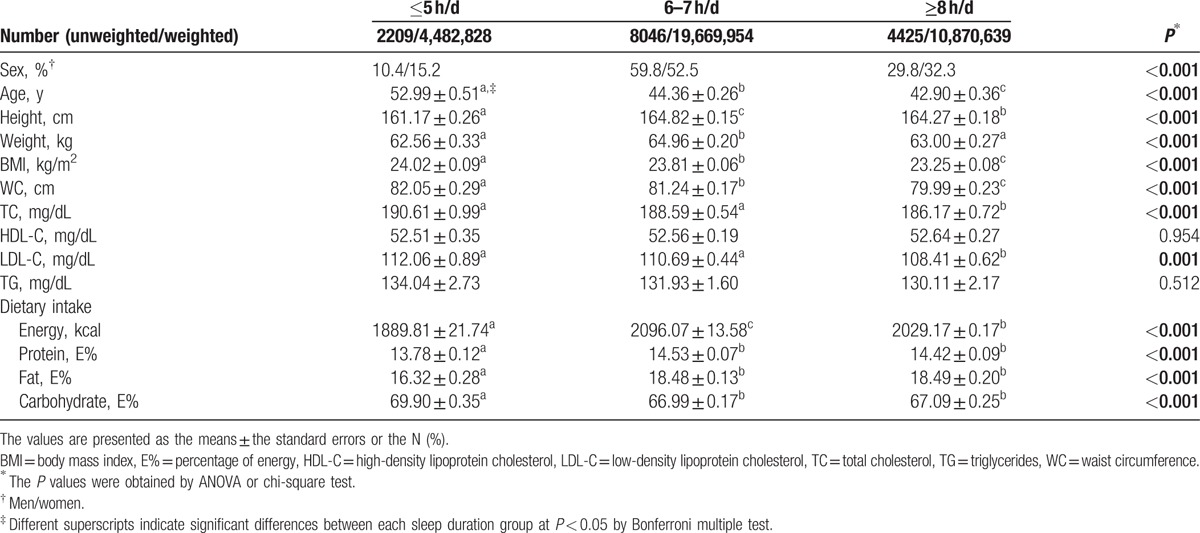
Anthropometric, blood lipid and dietary consumption of a Korean population by sleep duration.

However, participants with the shortest sleep duration (≤5 hours a day) consumed less dietary protein (*P* < 0.001) and fat (*P* < 0.001). Additionally, dietary CHO consumption was higher in the shortest sleep duration group (*P* < 0.001).

### 3.3 Effects of the interactions of sleep duration and dietary macronutrients consumption on obesity

**Figure 2:**
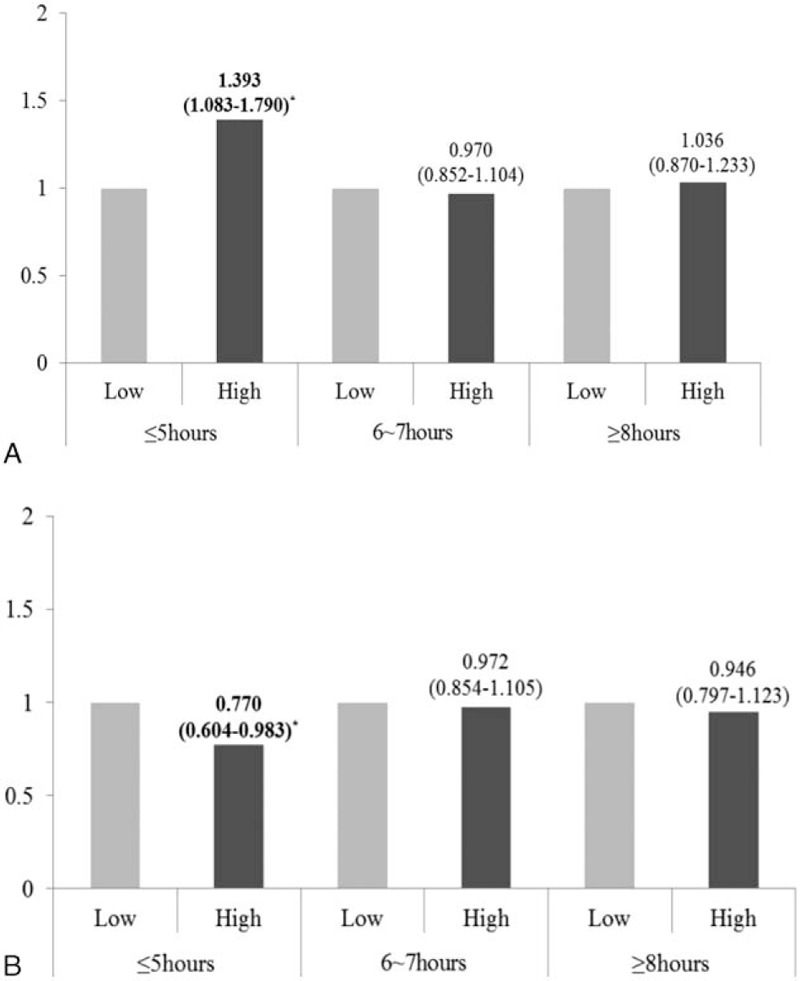
Effects of the interactions of sleep duration and dietary fat (A) and carbohydrate (B) consumption on obesity in a Korean population. Sleep durations were categorized as ≤5 hours, 6 to 7 hours, and ≥8 hours a day. ▪, low intake; ▪, high intake. The odds ratios (95% confidence intervals) and corresponding *P* values (^∗^*P* < 0.05) after adjusting for sex and age in the multiple logistic regression analysis are presented.

To analyze whether the interaction between sleep duration and dietary consumption affected the risks of obesity and dyslipidemia, a multivariable logistic regression analysis was performed after adjusting for age and sex. There was a significant interaction between sleep duration and dietary consumption only regarding the risk of obesity (*P* interaction < 0.001 for the consumption of all dietary macronutrients). However, there was no significant interaction between sleep duration and dietary consumption on the risk of dyslipidemia. Regarding the effects of sleep duration and dietary consumption on the risk of obesity, sleep duration significantly influenced the risk of obesity via dietary fat and CHO levels (Fig. 2). Of the participants with short sleep durations, the risk of obesity was higher in those with a high fat intake diet compared with those with a low fat intake [OR (95% CI) 1.393 (1.083–1.790), *P* < 0.005]. In contrast, the risk of obesity was lower in those with a low CHO intake [OR (95% CI) 0.770 (0.604–0.983), *P* < 0.005]. High fat and low CHO consumptions were confirmed to be associated with risk of obesity in the shortest sleep duration group (≤5 hours a day). There were no significant differences in the risk of obesity according to dietary fat or CHO consumption levels among those who slept 6 to 7 hours or ≥8 hours a day.

## 4 Discussion

The results of this study showed that sleep duration is associated with obesity and dyslipidemia-related indices and with dietary macronutrient consumption in the Korean population. Moreover, we confirmed the effects of the interactions between low sleep duration and dietary consumption such as fat and CHO on the risk of obesity in a relatively large sample of the population. However, there were no significant interaction effects between sleep duration and dietary consumption on the risk of dyslipidemia.

In this study, sleep duration was found to be associated with obesity and dyslipidemia-related indices, including BMI, WC, TC, and LDL-C, and these findings corroborate those of previous studies.^[2,3,5,6,8]^ However, this study revealed inverse relationships between sleep duration and obesity and dyslipidemia-related indices, and these findings are inconsistent with those of several studies,^[2,3,5,6]^ which have suggested a U-shaped association between sleep duration and these metrics. These differences can be explained by differences in the definitions of the sleep duration categories. For example, Grandner et al^[1]^ classified a short sleep duration as <5 to 6 hours a day and a long sleep duration as ≥9 hours a day, whereas another study classified ≤6 hours a day as a short sleep duration and ≥9 hours a day as a long sleep duration.^[14]^ Therefore, the variations in the definitions of sleep duration may have contributed to the different results identified by the studies.

Several studies have reported a significant association between dietary energy consumption and sleep duration.^[12,20–23]^ The result of our study also showed that subjects with a short sleep duration tended to have a decreased dietary energy consumption. These results differ from those of other studies, in which a short sleep duration was found to be associated with increased dietary energy consumption.^[20,21]^ Additionally, the present study observed that short sleep durations were associated with decreases in dietary fat and protein consumption, and the consumption of a large amount of CHO. These findings are partly consistent with previous studies, which have also shown that individuals with short sleep durations consume more energy-rich foods, particularly high-CHO foods, due to increased hunger and decreased satiety.^[22,23]^ However, 2 studies in Chinese populations^[12,21]^ reported contradictory results regarding the association between sleep duration and dietary consumption, even though both of these studies were performed in Asian populations.

Interestingly, dietary consumption, particularly dietary fat and CHO consumption, modified the effect of sleep duration on the risk of obesity, but not on the risk of dyslipidemia after adjusting for age and sex. Among only the participants who slept ≤5 hours a day, the risk of obesity was increased for those who consumed more fat and decreased for those who consumed more CHO. A previous study^[24]^ reported that children with short sleep durations are at an increased risk of overweight/obesity and that they consume more CHO. The results of other populations clearly cannot be generalized to the whole, and these findings are inconsistent with those of our study. However, greater proportions of dietary fat (compared with greater proportions of dietary CHO) can be interpreted as greater consumptions of energy-dense diets,^[25]^ which consequently result in an increase in the risk of obesity. Moreover, the consumption of highly fatty diets could adversely affect health outcomes, such as abdominal fat and dyslipidemia.^[26]^ In contrast, a higher dietary CHO proportion is related to a greater consumption of healthy foods, including fruits, vegetables, beans, and fiber-rich foods.^[27]^ Furthermore, most Koreans typically consume CHO-rich foods as staples of their diet, which means that the traditional diets of Koreans are higher in CHO and lower in fat. Therefore, this study may not be fully generalizable to Western populations. In addition, future studies are needed to confirm these findings in other populations.

Although the results of this study have several limitations regarding their interpretation, this study provides a basis for examining relatively large populations in terms of the effects of the interactions between sleep duration and dietary consumption patterns on the risks of obesity and dyslipidemia. These findings provide a foundation for future studies to examine whether the modification of sleep duration and dietary consumption delays the development of obesity and dyslipidemia. However, Leischik et al reported that the types of health problems explored in this study, such as obesity and dyslipidemia, are determined by different factors including genetics, medical care, national or global policy, empowerment (health literacy, education), socioeconomic status, and type of setting (environment, healthy cities), and also by health behavior (physical activity, nutrition, and sleep condition).^[28]^ In particular, with the rapid and unplanned urbanization and industrialization emerging from globalization, various changes in the patterns of physical activity, and also diet have occurred.^[25,29,30]^ These changes in physical activity are associated with an increase in sedentary behaviors—using motorized transportation, having sedentary occupations, spending leisure time watching TV, playing computer games, and checking mobile phones.^[25,31]^ Additionally, the design of a city's built environment is one of the factors affecting the time spent on sedentary behavior.^[31,32]^ The increase in sedentary behaviors has been reported to be associated with cardiometabolic problems including obesity, type 2 diabetes mellitus, metabolic syndrome, and cardiovascular disease.^[25,29–31,33–35]^ Moreover, health is influenced by disparities in socioeconomic status, such as differences in education level, household income, and place of residence.^[34,36,37]^ A recent study^[37]^ reported that various health policies have been able to decrease the disparities in socioeconomic status, with the greatest reduction associated with physical activity-related policies. Therefore, as mentioned by Leischik et al, future studies will need to consider multidimensional factors including the level of physical activity and the types and qualities of foods composing the diet, and also sleep status, in relation to the risk of obesity and dyslipidemia.

## References

[1] Grandner MA Chakravorty S Perlis ML Habitual sleep duration associated with self-reported and objectively determined cardiometabolic risk factors. *Sleep Med* 2014; 15:42–50. 24333222 10.1016/j.sleep.2013.09.012 PMC3947242

[2] Marshall NS Glozier N Grunstein RR Is sleep duration related to obesity? A critical review of the epidemiological evidence. *Sleep Med Rev* 2008; 12:289–298. 18485764 10.1016/j.smrv.2008.03.001

[3] Buxton OM Marcelli E Short and long sleep are positively associated with obesity, diabetes, hypertension, and cardiovascular disease among adults in the United States. *Soc Sci Med* 2010; 71:1027–1036. 20621406 10.1016/j.socscimed.2010.05.041

[4] Cappuccio FP D’Elia L Strazzullo P Quantity and quality of sleep and incidence of type 2 diabetes: a systematic review and meta-analysis. *Diabetes Care* 2010; 33:414–420. 19910503 10.2337/dc09-1124 PMC2809295

[5] Kinuhata S Hayashi T Sato KK Sleep duration and the risk of future lipid profile abnormalities in middle-aged men: the Kansai Healthcare Study. *Sleep Med* 2014; 15:1379–1385. 25220668 10.1016/j.sleep.2014.06.011

[6] Lee JA Park HS Relation between sleep duration, overweight, and metabolic syndrome in Korean adolescents. *Nutr Metab Cardiovasc Dis* 2014; 24:65–71. 24188647 10.1016/j.numecd.2013.06.004

[7] Ford ES Habitual sleep duration and predicted 10-year cardiovascular risk using the pooled cohort risk equations among US adults. *J Am Heart Assoc* 2014; 3:e001454. 25468656 10.1161/JAHA.114.001454 PMC4338737

[8] Xiao Q Arem H Moore SC A large prospective investigation of sleep duration, weight change, and obesity in the NIH-AARP Diet and Health Study cohort. *Am J Epidemiol* 2013; 178:1600–1610. 24049160 10.1093/aje/kwt180 PMC3842900

[9] Klop B Elte JW Cabezas MC Dyslipidemia in obesity: mechanisms and potential targets. *Nutrients* 2013; 5:1218–1240. 23584084 10.3390/nu5041218 PMC3705344

[10] Lopez-Miranda J Williams C Lairon D Dietary, physiological, genetic and pathological influences on postprandial lipid metabolism. *Br J Nutr* 2007; 98:458–473. 17705891 10.1017/S000711450774268X

[11] St-Onge MP The role of sleep duration in the regulation of energy balance: effects on energy intakes and expenditure. *J Clin Sleep Med* 2013; 9:73–80. 23319909 10.5664/jcsm.2348 PMC3525993

[12] Weiss A Xu F Storfer-Isser A The association of sleep duration with adolescents’ fat and carbohydrate consumption. *Sleep* 2010; 33:1201–1209. 20857867 10.1093/sleep/33.9.1201 PMC2938861

[13] The Korea Centers for Disease Control and Prevention. The fifth Korean National Health and Nutrition Survey. 2010–2012. Available at: https://knhanes.cdc.go.kr/ Accessed May 12, 2016.

[14] Knutson KL Sleep duration and cardiometabolic risk: a review of the epidemiologic evidence. Best Practice and Research. *Clin Endocrinol Metab* 2010; 24:731–743. 10.1016/j.beem.2010.07.001 PMC3011978 21112022

[15] Gallicchio L Kalesan B Sleep duration and mortality: a systematic review and meta-analysis. *J Sleep Res* 2009; 18:148–158. 19645960 10.1111/j.1365-2869.2008.00732.x

[16] Oh SW Shin SA Yun YH Cut-off point of BMI and obesity-related comorbidities and mortality in middle-aged Koreans. *Obesity Res* 2004; 12:2031–2040. 10.1038/oby.2004.254 15687405

[17] Friedewald WT Levy RI Fredrickson DS Estimation of the concentration of low-density lipoprotein cholesterol in plasma without use of the preparative ultracentrifuge. *Clin Chem* 1972; 18:499–502. 4337382

[18] Korean Society of Lipidology, Atherosclerosis The Treatment Guidelines of Dyslipimia, 2nd ed. Seoul:Korean Society of Lipidology and Atherosclerosis; 2009.

[19] Kim DW Song S Lee JE Reproducibility and validity of an FFQ developed for the Korea National Health and Nutrition Examination Survey (KNHANES). *Public Health Nutr* 2014; 28:1–9. 10.1017/S1368980014001712 PMC10271806 25167205

[20] Patterson RE Emond JA Natarajan L Short sleep duration is associated with higher energy intake and expenditure among African-American and non-Hispanic white adults. *J Nutr* 2014; 144:461–466. 24523490 10.3945/jn.113.186890 PMC3952622

[21] Shi Z McEvoy M Luu J Dietary fat and sleep duration in Chinese men and women. *Int J Obesity* 2008; 32:1835–1840. 10.1038/ijo.2008.191 18982012

[22] Nedeltcheva AV Kilkus JM Imperial J Sleep curtailment is accompanied by increased intake of calories from snacks. *Am J Clin Nutr* 2009; 89:126–133. 19056602 10.3945/ajcn.2008.26574 PMC2615460

[23] Markwald RR Melanson EL Smith MR Impact of insufficient sleep on total daily energy expenditure, food intake, and weight gain. *Proc Natl Acad Sci USA* 2013; 110:5695–5700. 23479616 10.1073/pnas.1216951110 PMC3619301

[24] Firouzi S Poh BK Ismail MN Sleep habits, food intake, and physical activity levels in normal and overweight and obese Malaysian children. *Obesity Res Clin Pract* 2014; 8:e70–e78. 10.1016/j.orcp.2012.12.001 24548579

[25] World Health Organisation. Diet, nutrition and the prevention of chronic diseases. 2003. Available at: http://www.who.int/dietphysicalactivity/publications/trs916/en/ Accessed September 12, 2016,

[26] Melanson EL Astrup A Donahoo WT The relationship between dietary fat and fatty acid intake and body weight, diabetes, and the metabolic syndrome. *Ann Nutr Metab* 2009; 55:229–243. 19752544 10.1159/000229004

[27] Roberts SB High-glycemic index foods, hunger, and obesity: is there a connection? *Nutr Rev* 2000; 58:163–169. 10885323 10.1111/j.1753-4887.2000.tb01855.x

[28] Leischik R Dworrak B Strauss M Plasticity of Health. *German J Med* 2016; 1:1–17.

[29] Blair SN Physical inactivity: the biggest public health problem of the 21st century. *Br J Sports Med* 2009; 43:1–2. 19136507

[30] World Health Organization. Global Strategy on Diet, physical Activity and Health. 2004. Available at: http://www.who.int/dietphysicalactivity/strategy/eb11344/strategy_english_ web.pdf Accessed September 12, 2016.

[31] Young DR Hivert M-F Alhassan S Sedentary Behavior and Cardiovascular Morbidity and Mortality. A Science Advisory From the American Heart Association. *Circulation* 2016; 134:e262–e279. 27528691 10.1161/CIR.0000000000000440

[32] World Health Organization. Healthy Cities. 2016. Available at: http://www.euro.who.int/en/health-topics/environment-and-health/urban-health/activities/healthy-cities Accessed September 12, 2016.

[33] Leischik R Foshag P Strauss M Physical activity, cardiorespiratory fitness and carotid intima thickness: sedentary occupation as risk factor for atherosclerosis and obesity. *Eur Rev Med Pharmacol Sci* 2015; 19:3157–3168. 26400517

[34] World Health Organisation. Evaluation in health promotion. Principles and perspectives. World Health Organisation, 2001. Available at: http://www.euro.who.int/en/publications/abstracts/evaluation-in-health-promotion.-principles-and-perspectives Accessed September 12, 2016.

[35] Sperling LS Mechanick JI Neeland IJ The CardioMetabolic Health Alliance: working toward a new care model for the metabolic syndrome. *J Am Coll Cardiol* 2015; 66:1050–1067. 26314534 10.1016/j.jacc.2015.06.1328

[36] Shrivastava U Misra A Gupta R Socioeconomic factors relating to diabetes and its management in India. *J Diabetes* 2016; 8:12–23. 26019052 10.1111/1753-0407.12316

[37] Orr MG Kaplan GA Galea S Neighbourhood food, physical activity, and educational environments and black/white disparities in obesity: a complex systems simulation analysis. *J Epidemiol Commun Health* 2016; 70:862–867. 10.1136/jech-2015-205621 27083491

